# Infratentorial white matter integrity as a potential biomarker for post-stroke aphasia

**DOI:** 10.1093/braincomms/fcaf174

**Published:** 2025-05-06

**Authors:** Ben Zhang, Caroline Schnakers, Kevin Xing-Long Wang, Jing Wang, Sharon Lee, Henry Millan, Melissa Howard, Emily Rosario, Zhong Sheng Zheng

**Affiliations:** Casa Colina Hospital and Centers for Healthcare, Research Institute, Pomona, CA 91767, USA; Casa Colina Hospital and Centers for Healthcare, Research Institute, Pomona, CA 91767, USA; Psychology Department, University of California, Los Angeles, CA 90095, USA; Casa Colina Hospital and Centers for Healthcare, Research Institute, Pomona, CA 91767, USA; Casa Colina Hospital and Centers for Healthcare, Research Institute, Pomona, CA 91767, USA; Casa Colina Hospital and Centers for Healthcare, Research Institute, Pomona, CA 91767, USA; Casa Colina Hospital and Centers for Healthcare, Research Institute, Pomona, CA 91767, USA; Casa Colina Hospital and Centers for Healthcare, Research Institute, Pomona, CA 91767, USA; Casa Colina Hospital and Centers for Healthcare, Research Institute, Pomona, CA 91767, USA

**Keywords:** stroke, aphasia, DTI, cerebellum, WAB-R

## Abstract

Traditionally, neuroimaging studies of post-stroke aphasia focus on supratentorial brain regions related to language function and recovery. However, stroke-induced lesions often distort these areas, posing a challenge for neuroimaging analyses aimed at identifying reliable biomarkers. This study seeks to explore alternative biomarkers in regions less affected by direct stroke damage, such as white matter regions below the tentorium, to overcome these methodological limitations. Diffusion tensor imaging was accomplished on 55 participants with chronic post-stroke aphasia. Focusing on regions below the tentorium, correlations were analysed between Western Aphasia Battery-Revised scores and average fractional anisotropy values. The volume of intersection between each participant's lesion and their left arcuate fasciculus was also analysed for correlations with Western Aphasia Battery-Revised scores as well. Linear regression analyses were then conducted using regions showing significant correlations as univariate predictors. After applying multiple comparisons corrections, we found that average fractional anisotropy in the middle cerebellar peduncle was positively correlated with aphasia quotient (*P* = 0.004), spontaneous speech (*P* = 0.005), auditory verbal comprehension (*P* = 0.004), naming and word finding (*P* = 0.005) and repetition (*P* = 0.013). Average fractional anisotropy in the left inferior cerebellar peduncle positively correlated with spontaneous speech (*P* = 0.018) and auditory verbal comprehension (*P* = 0.018). Average fractional anisotropy in the left corticospinal tract positively correlated with aphasia quotient (*P* = 0.019) and spontaneous speech (*P* = 0.005). The volume of intersection between the left arcuate fasciculus and participant lesion was negatively correlated with aphasia quotient (*P* = 0.014) and repetition (*P* = 0.002). Through linear regression analyses, average fractional anisotropy of the middle cerebellar peduncle significantly predicted aphasia quotient and all subscores. Average fractional anisotropy of the left inferior cerebellar peduncle significantly predicted all scores except repetition. Average fractional anisotropy of the left corticospinal tract significantly predicted all scores except for auditory verbal comprehension. The volume of intersection between the left arcuate fasciculus and lesions significantly predicted all scores except for auditory verbal comprehension. These findings underscore the potential of infratentorial white matter regions as biomarkers of aphasia severity, encompassing overall and specific subdomain impairment. By shifting the focus to below the tentorium, it becomes possible to find more robust targets for further research and therapeutic interventions. This approach is not only able to sidestep analytical complications posed by cortical lesions, it also opens new doors for understanding complex cerebellar mechanisms that underlie language function and recovery post-stroke.

## Teaser

Zhang *et al*. investigate post-stroke aphasia in relation to infratentorial white matter integrity, reporting that fractional anisotropy in the middle cerebellar peduncle, left inferior cerebellar peduncle, and left corticospinal tract correlate with aphasia severity. This highlights cerebellar white matter as a potential non-supratentorial biomarker for language recovery.

## Graphical Abstract

**Graphical Abstract:**
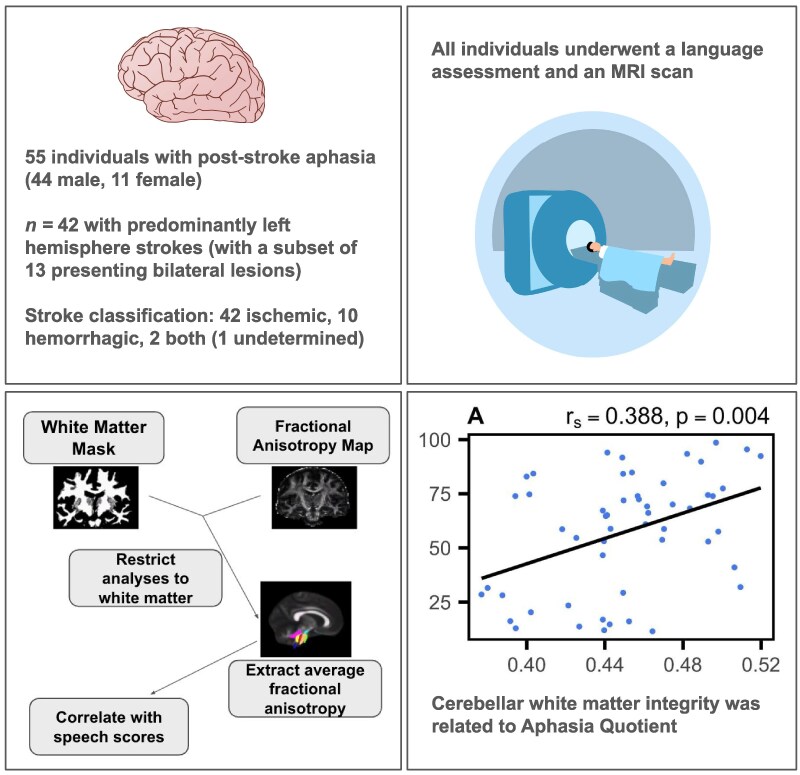

## Introduction

Aphasia is a complex neurological condition that affects millions globally, characterized by deficits in language production and comprehension commonly resulting from stroke.^1^ Research in post-stroke aphasia (PSA) has primarily focused on supratentorial regions, including cortical language areas (e.g. Broca's and Wernicke's areas) and associated white matter tracts (e.g. arcuate fasciculus).^1-5^ However, when dealing with brain-distorting lesions resulting from supratentorial stroke, this limited perspective leads to methodological challenges in research and clinical assessments. While exploring cortical contributions to aphasia has provided valuable insights, the significance of white matter integrity below the tentorium (infratentorium) and its relationship to PSA has often been overlooked.^3,4^

Language processing and speech production rely on an extensive network that exceeds the well-known Broca's and Wernicke's areas, engaging a myriad of cortical, subcortical and cerebellar regions.^6-10^ Recent research has demonstrated the importance of the cerebro-cerebellar network, with the cerebellum—situated beneath the tentorium—playing a crucial role.^11^ The cerebellum is consistently implicated in language function, playing a role in motor components such as articulation, as well as non-motor aspects of language such as semantics.^12^ To touch on motor components, neuroimaging studies have shown activation of representations of motor effectors in the cerebellum during tongue movement or articulation.^11,12^ Furthermore, early clinical evidence of perceptual deficits in temporal discrimination tasks associated with cerebellar pathology shows how the cerebellum partially functions as an ‘internal clock’ for motor and perceptual timing. Consistent with this, dysfunction in infratentorial regions appears to impair temporal aspects of speech perception, compromising fluent speech..^8,9,11-13^ Non-motor aspects of language, such as phoneme identification and speech distortion adaptation, have also been shown to be correlated with the cerebellum, particularly the right Crus I.^11,13^ Many studies also report semantic deficits in patients with cerebellar dysfunction.^12^ Evidence suggests that the right lateral cerebellum plays a role in non-motor functions, specifically semantic processing. PET and functional magnetic resonance imaging (fMRI) studies show activation of the fronto-parietal cortex as well as the contralateral right cerebellar hemisphere during word generation tasks with minimized motor demands, indicating a cognitive role beyond speech production.^9,10^ Transcranial magnetic stimulation studies examining disruption of the right cerebellum saw impaired semantic fluency.^9,13^ Additionally, observed cognitive deficits such as verbal learning and working memory may have an anatomical basis in the reciprocal fibre systems connecting the cerebellum with the cerebral association cortex.^13^

Following a brain injury, white matter fibres may shift, causing disconnections that necessitate neural reorganization and adaptation.^14^ This reorganization process is critical for recovery from aphasia, as demonstrated by research linking changes in white matter integrity to improvements in language functions.^15^ The relevance of white matter pathways to speech challenges, including issues with repetition (REP), comprehension and naming, has also been increasingly recognized.^16-18^ Despite these insights, however, the role of infratentorial white matter integrity in PSA remains understudied. This gap in understanding is especially concerning due to the issues posed during analysis of supratentorial regions in PSA patients, as lesions often distort and complicate accurate delineation of white matter tracts.

Our study focuses on addressing this gap by exploring the association between white matter integrity below the tentorium and language function in patients with chronic PSA. By focusing on infratentorial regions, which are generally less affected in PSA, we aim to identify potential biomarkers that could lead to more accessible and clinically reliable measures of PSA.

Utilizing diffusion tensor imaging (DTI), a pivotal method for evaluating white matter integrity, we investigated the microstructural properties of infratentorial white matter tracts alongside their relationship to language function as measured by the Western Aphasia Battery-Revised (WAB-R).^19,20^ As many DTI studies have been supratentorially focused, our approach opens up new avenues for understanding intricate changes in language-related white matter following stroke by circumventing challenges from cortical damage.^21-23^

The results of this study could have significant implications for both research and clinical practice in PSA. By investigating the relationships between white matter integrity in infratentorial brain regions and language function, we may help provide a more encompassing understanding of neural correlates of PSA, as well as potentially offer new and more robust targets for therapeutic interventions and prognostic indicators.

## Materials and methods

### Participants

**Figure 1:**
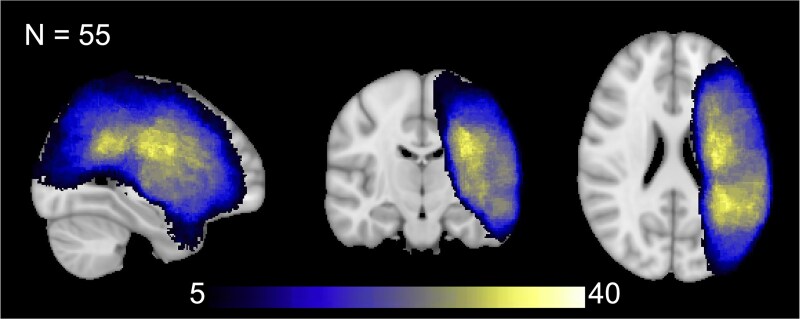
**Group lesion overlap.** The group lesion map (*N* = 55) indicates areas of stroke damage shared by participants. Lesion overlap was most pronounced in the left perisylvian regions, particularly within the left superior longitudinal fasciculus. The colour bar reflects the number of participants with overlapping lesions in each region, ranging from 5 (minimum threshold for display) to 40 participants.

The dataset (behavioural and diffusion-weighted data) used in this analysis was derived from the pre-intervention (i.e. baseline) data of two previous clinical trials that investigated the efficacy of transcranial direct current stimulation in treating PSA. While the original trials included both pre- and post-intervention timepoints, only the pre-intervention data were utilized in the current study. This approach allowed for the combination of data from both trials, resulting in a larger dataset for analysis. The sample included 57 participants with chronic PSA, excluding two participants with right hemisphere-only lesions for a final cohort of 55. This final cohort consisted of predominantly left hemisphere strokes (*n* = 42), with a subset of participants presenting bilateral lesions (*n* = 13). The group lesion overlap of the patients is shown in Fig. 1. The inclusion criteria included individuals with PSA (diagnosed by a Neurologist or Speech/Language Therapist) without a history of other neurological disorders, were at least 18 years or older, 6 months post-stroke and English proficient. Clinical as well as demographic data is included in Table 1. This study was approved by the Institutional Review Board at Casa Colina Hospital and Centers for Healthcare, and all participants provided written informed consent prior to enrolling.

**Table 1:** Patient demographics and clinical information

Variable	Num or mean	Range
Age (years)	60.3 ± 3.67	21–83
TSI (days)	1440.3 ± 344.54	316–5945
WAB-R score		
AQ	57.32 ± 7.23	11.5–98.6
SS	10.49 ± 1.50	0–20
AVC	7.53 ± 0.54	2.35–10
NW	5.09 ± 0.88	0–9.6
Repetition	5.55 ± 0.91	0–10
Gender		
Male	44	
Female	11	
Stroke type (ischaemic/haemorrhagic/both/unsure)		
Ischaemic	42	
Haemorrhagic	10	
Both	2	
Unsure	1	
Fluency (fluent/non-fluent)		
Fluent	25	
Non-fluent	30	
Lesion size (thousands of voxels)	178.98 ± 35.70	1.45–542.44
Lesion type		
Left hemisphere	42	
Bilateral		
Bilateral cortical	5	
Bilateral subcortical	3	
Left cortical + right subcortical	5	

### Language assessment

All participants underwent aphasia assessment using the WAB-R. This comprehensive assessment evaluates language capabilities across four dimensions: spontaneous speech (SS), auditory verbal comprehension (AVC), naming and word finding (NW) and REP. Additionally, the WAB-R provides an overall aphasia quotient (AQ) [range 0–100], which encompasses the four subscores and provides a measure of aphasia severity, with higher scores indicating better language performance.

### MRI acquisition

A 3T Siemens Magnetom Verio scanner was used to acquire MRI data. Using T1-weighted Magnetization Prepared Rapid Gradient Echo (MPRAGE) sequence [repetition time (TR) = 2300 ms; echo time (TE) = 2 ms; flip angle = 9°; field of view (FOV) = 230 mm; slice thickness = 1 mm with no gap; number of slices = 160; matrix size = 224 × 224], a high resolution 3D anatomical scan was collected. Diffusion-weighted imaging was then performed with the following parameters: TR = 10 900 ms; TE = 95 ms; slice thickness = 2 mm with no gap; number of slices = 82; matrix size = 122 × 122. Diffusion-sensitizing gradients were applied along 64 non-collinear directions (*b* = 1000 s/mm^2^). Five images without no diffusion weighting (*b* = 0) were also obtained.

### Preprocessing and lesion identification

Initially, T1-weighted images were subjected to bias-field correction utilizing FMRIB Software Library (FSL) tools (http://www.fmrib.ox.ac.uk/fsl). Following this, the optimized brain extraction for pathological brains (optiBET) tool was employed for brain extraction.^24^ Stroke lesions were delineated using the automated segmentation tool LINDA (Lesion Identification with Neighborhood Data Analysis), which processes the patients' T1-weighted images.^25^ Subsequently, lesion masks generated by LINDA were manually refined as necessary. For standard space alignment of individual T1-weighted lesion masks [Montreal Neurological Institute (MNI) 1 mm], we utilized advanced normalization tools.^26^ These standardized lesion masks were then binarized and aggregated to create a group lesion map, shown in Fig. 1. The preprocessing of DWI data was conducted with DTIPrep, which served to identify and eliminate artifact-laden volumes, alongside corrections for eddy currents and head motion via affine registration to the average *b* = 0 reference image.^27^ The skull stripping of the *b* = 0 image was accomplished using the brain extraction tool (BET).^28^

### DTI analysis

After preprocessing, diffusion tensors were calculated at each voxel of the brain using the FSL's diffusion toolbox. From these tensors, fractional anisotropy (FA) maps were computed for each participant.

**Figure 2:**
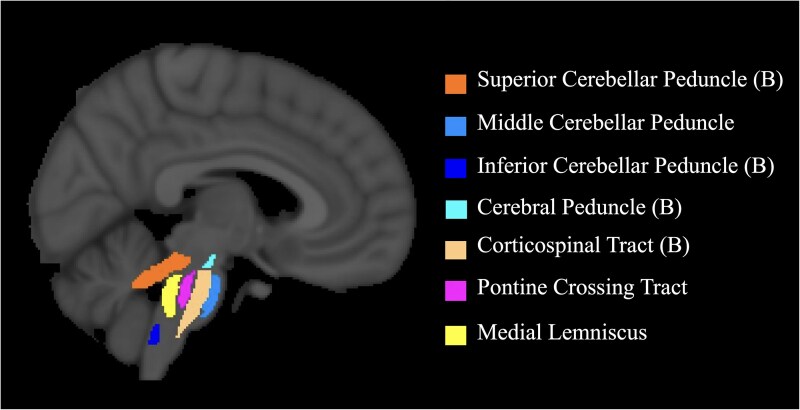
**Infratentorial white matter regions of interest.** Sagittal view displaying infratentorial white matter labels from the Johns Hopkins White Matter Atlas. (B) = Bilateral structures.

We employed a region of interest (ROI) approach using the John Hopkins University (JHU) white matter atlas (https://neurovault.org/collections/264/). Due to the extensiveness and heterogeneity of the lesions, which primarily affected regions above the tentorium, accurate delineation of supratentorial ROIs proved challenging. Therefore, we focused our investigation on infratentorial regions, which were predominantly intact across all patients. Twelve ROIs were selected (Fig. 2): middle cerebellar peduncle (MCP), pontine crossing tract, bilateral medial lemniscus (ML), bilateral cerebral peduncle (CP), bilateral corticospinal tract (CST), bilateral superior cerebellar peduncle (SCP) and bilateral inferior cerebellar peduncle (ICP). Applying nonlinear registration between the standard space (FMRIB58_FA) and patients' diffusion space, the standard ROIs were transformed into the latter. DTI analyses were conducted in native space with nonlinear registration to account for susceptibility-induced distortions and resolution differences inherent to diffusion-weighted imaging. Unlike structural MRI, DTI is particularly prone to geometric misalignments due to subject motion, eddy currents and magnetic field inhomogeneities,^27,29^ requiring nonlinear transformations to achieve accurate spatial alignment. While supratentorial stroke lesions can introduce distortions during nonlinear registration, our focus on infratentorial white matter ensures that these effects do not compromise the regions of interest. Before analysis of data, ROIs were examined visually for accuracy and corrected manually if required. A whole-brain white matter segmentation mask was generated for each patient to intersect with the ROIs, ensuring that only white matter voxels were included. The average FA of each ROI was calculated for each patient, providing a measure of white matter integrity. In the Supplementary material, we have included code used for DTI analyses. FA was selected as the primary metric in this study due to its broad sensitivity to white matter integrity and its practicality in exploratory analyses. As a composite measure incorporating aspects of both radial and axial diffusion, FA provides a single, robust marker that can reflect changes in fibre organization, density and myelination.^17^ Additionally, FA is widely used in both research and clinical contexts, facilitating comparability across studies. While axial diffusivity (AD) and radial diffusivity (RD) may offer more specific mechanistic insights into axonal integrity and myelination, their interpretation is highly context-dependent (e.g. crossing fibres, gliosis and partial volume effects) and requires more targeted hypotheses.^17,23^

### Statistical analysis

To assess baseline differences among participants, we initially performed Spearman's rank correlations between the mean FA of each ROI, WAB-R scores, age, and time since injury (TSI). Given that neither age nor TSI exhibited significant correlations with the variables of interest, no covariates were included in subsequent correlation analyses. All statistical analyses were done using JASP version 0.19.3 (https://jasp-stats.org/).

Spearman's rank correlations were then conducted, exploring the relationship between baseline average FA in infratentorial regions and WAB-R scores after stroke. For comparative purposes, we also analysed Spearman's correlations between left arcuate lesion volume and WAB-R scores, as most participants had lesions affecting this canonical language structure. While white matter integrity measures like FA could be informative, especially in infratentorial regions (relatively spared in PSA), the extensive lesions in our cohort precluded reliable brain registration in regions above the tentorium. This limitation necessitated that our supratentorial focus be on lesion volume. The Benjamini–Hochberg method (FDR = 0.05) was utilized for multiple comparisons correction.

Building on these findings, univariate linear regression analyses were conducted to pinpoint which significant white matter regions served as reliable predictors of WAB-R scores. A univariate linear regression analysis between the arcuate lesion volumes and WAB-R scores was carried out as well for the purpose of comparison. Average FA values of the MCP, L-ICP and L-CST, along with the left arcuate lesion volume, were used as independent variables, and WAB-R scores (AQ, SS, AVC, NWF and REP) were dependent variables.

## Results

**Figure 3:**
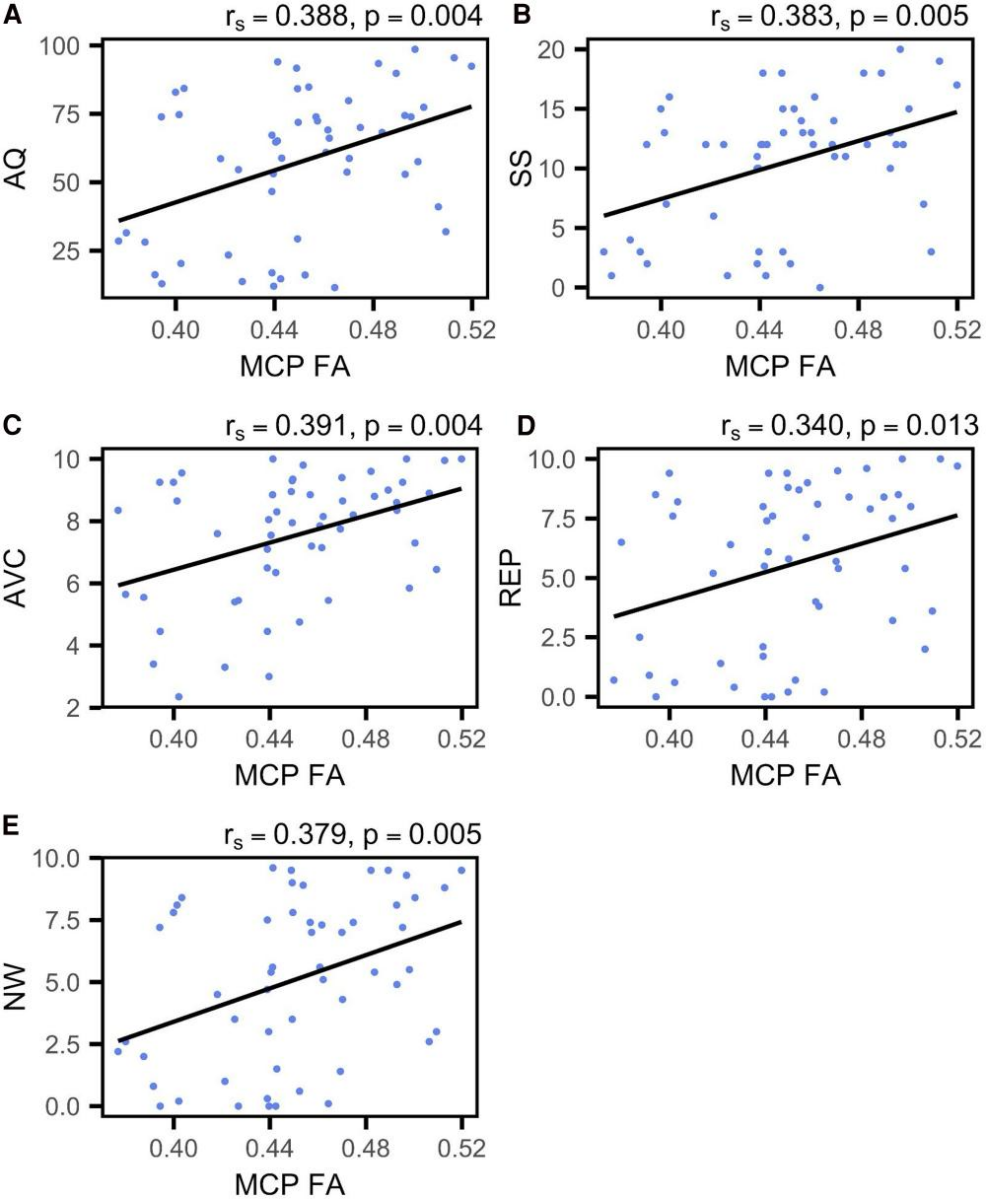
**Middle cerebellar peduncle correlations with WAB-R scores.** (**A–E**) Scatterplots indicate Spearman's correlation between the average FA of the MCP and WAB-R scores (*N* = 55). Plots are shown for (**A**) AQ, (**B**) SS, (**C**) AVC, (**D**) REP and (**E**) NW. Each data point represents an individual participant.

**Figure 4:**
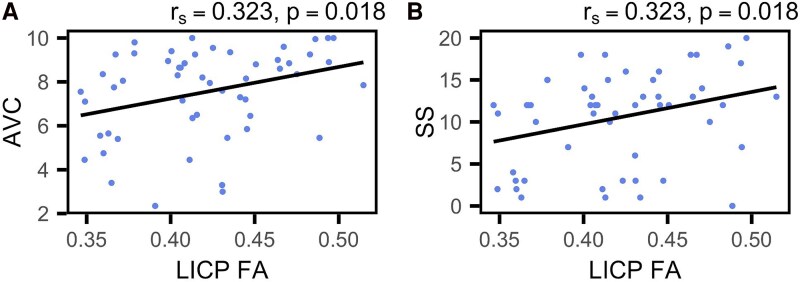
**Left Inferior cerebellar peduncle correlations with WAB-R scores.** (**A** and **B**) Scatterplots indicate Spearman's correlation between the average FA of the L-ICP and WAB-R scores (*N* = 55). Plots are shown for (**A**) AVC, and (**B**) SS. Each data point represents an individual participant.

**Figure 5:**
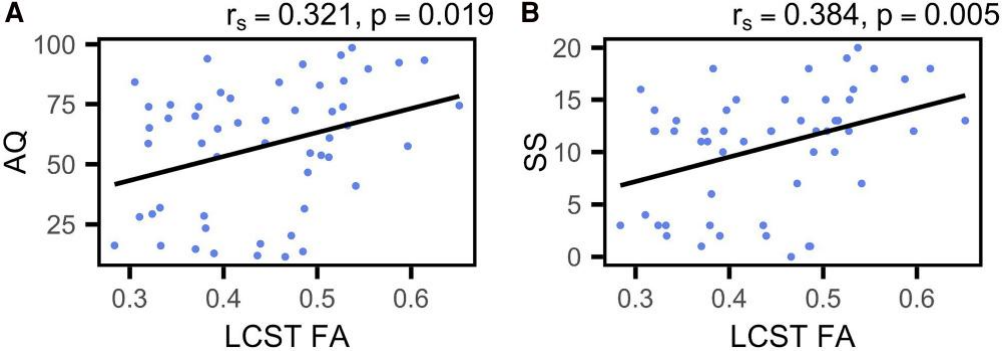
**Left corticospinal tract correlations with WAB-R scores.** (**A** and **B**) Scatterplots indicate Spearman's correlation between the average FA of the L-CST and WAB-R scores (*N* = 55). Plots are shown for (**A**) AQ, and (**B**) SS. Each data point represents an individual participant.

The group lesion overlap map portrayed damage mostly focused in the left perisylvian regions, with the largest overlap occupying the left superior longitudinal fasciculus (see Fig. 1), which has substantial anatomical overlap with the left arcuate fasciculus. Following correction for multiple comparisons, significant correlations were observed between the mean FA in the MCP and all WAB-R scores. Specifically, correlations were found with the AQ (*r*_s_ = 0.388, *P* = 0.004), SS (*r*_s_ = 0.383, *P* = 0.005), AVC (*r*_s_ = 0.391, *P* = 0.004), NW (*r*_s_ = 0.379, *P* = 0.005) and REP (*r*_s_ = 0.340, *P* = 0.013). Similarly, FA in the left ICP (L-ICP) correlated with SS (*r*_s_ = 0.323, *P* = 0.018) and AVC (*r*_s_ = 0.323, *P* = 0.018). Additionally, FA in the left CST (L-CST) correlated with AQ (*r*_s_ = 0.321, *P* = 0.019) and SS (r_s_ = 0.384, *P* = 0.005). Graphical representations of these correlations are presented in Figs 3–5. For the sake of comparison, we found that after multiple comparisons correction, the left arcuate lesion volume correlated with WAB-R scores as well, specifically AQ (*r*_s_ = −0.352, *P* = 0.014) and REP (*r*_s_ = −0.433, *P* = 0.002; see Supplemental Results and Supplementary Fig. 1).

Refer to Supplementary Table 2 for Spearman's correlations between the average FA of all 12 infratentorial ROI's, as well as the left arcuate lesion volume. Notably, the average FA of the MCP showed strong correlations with the average FA of multiple infratentorial tracts (*P* < 0.001), and the average FA in the L-CST negatively correlated with arcuate fasciculus lesion volume (*P* < 0.001).

To assess the predictive value of FA in specific white matter tracts on aphasia severity, we followed our correlation analyses with linear regression analyses (Table 2). WAB-R scores served as dependent variables, while FA values in the MCP, L-CST and L-ICP were used as independent variables. FA in the MCP emerged as a significant univariate predictor for all WAB-R scores: AQ (*P* = 0.003), SS (*P* = 0.003), AVC (*P* = 0.003), NW (*P* = 0.005) and REP (*P* = 0.017). FA in the L-ICP significantly predicted four out of five WAB-R scores (except for REP): AQ (*P* = 0.037), SS (*P* = 0.023), AVC (*P* = 0.019) and NW (*P* = 0.028). Similarly, FA in the L-CST demonstrated significant predictive value for the following measures: AQ (*P* = 0.014), SS (*P* = 0.005), NW (*P* = 0.033) and REP (*P* = 0.048). The left arcuate lesion volume showed statistical significance as a predictor of AQ (*t*_53_ = −2.599, *P* = 0.013), SS (*t*_53_ = −2.198, *P* = 0.033), NW (*t*_53_ = −2.171, *P* = 0.035) and REP (*t*_53_ = −3.366, *P* = 0.002; see Supplementary Table 1).

**Table 2:** Linear regression results

Dependent variable	Independent variable	*t*	*P*-value
AQ			
Model 1	MCP	3.613	0.003*
Model 2	L-CST	2.543	0.014*
Model 3	L-ICP	2.145	0.037*
Model 4	MCP	2.272	0.028*
	Left arcuate lesion volume	−1.807	0.077
Model 5	L-CST	1.029	0.309
	Left arcuate lesion volume	−1.794	0.08
Model 6	L-ICP	1.64	0.108
	Left arcuate lesion volume	−2.486	0.017*
SS			
Model 1	MCP	3.166	0.003*
Model 2	L-CST	2.907	0.005*
Model 3	L-ICP	2.338	0.023*
AVC			
Model 1	MCP	3.117	0.003*
Model 2	L-CST	1.81	0.076
Model 3	L-ICP	2.424	0.019*
NW			
Model 1	MCP	2.946	0.005*
Model 2	L-CST	2.193	0.033*
Model 3	L-ICP	2.263	0.028*
REP			
Model 1	MCP	2.464	0.017*
Model 2	L-CST	2.03	0.048*
Model 3	L-ICP	1.018	0.314

^*^Indicates a significant *P*-value.

While controlling for damage to the arcuate fasciculus (left arcuate lesion volume) within our linear regression models, we found that MCP retained significance as a predictor of AQ (*t*_53_ = 2.272, *P* = 0.028) while L-CST and L-ICP were no longer significant.

## Discussion

Our study aimed to explore the potential significance of infratentorial white matter regions in PSA by investigating the relationship between language performance and infratentorial white matter integrity. This approach expands upon traditional focuses on cortical language areas and supratentorial white matter tracts, offering a unique advantage in that it is less affected by PSA lesions, which predominantly occur in supratentorial areas. By examining regions that are typically preserved in PSA, we provide a more consistent basis for assessing language function across patients with varying lesion profiles. Our findings can be summarized as follows: (i) mean FA values of the MCP, L-ICP, L-CST and also left arcuate fasciculus lesion volume were positively correlated with Western Aphasia Battery-Revised (WAB-R) scores; (ii) These regions demonstrated significance as univariate predictors of WAB-R scores in linear regression models.

FA, a measurement for the directionality of white matter microstructures such as myelin, microtubules and axons, acts as a proxy for white matter integrity.^29^ Lower levels of FA have been found to be associated with worse behavioural and motor outcomes following brain injury.^30-33^ Better baseline integrity of the MCP, L-ICP and L-CST following PSA could highlight stronger communication between the cerebellum and other brain regions involved in language processing. Strengthened connections in these three regions could be associated with improved motor coordination and speech production, resulting in increased AQ and accompanying subscores.^34,35^ Furthermore, the cerebellum is also involved in higher-order linguistic processes such as grammatical processing, lexical access and verbal working memory—aspects commonly impaired in aphasia.^36,37^

The MCP is the major afferent pathway to the cerebellum—originating from the pontine nuclei, which integrate information from various cortical areas to establish the cortico-ponto-cerebellar system.^38^ Inputs from sensory, motor and associative areas of the cerebral cortex are directed to the ipsilateral pontine nuclei, which in turn project to the contralateral cerebellar cortex through the MCP. Hence, the MCP is crucially involved in cognitive function and motor coordination.^37,39^ With higher FA in the MCP reflecting better structural white matter integrity, we speculate that in turn it would be associated with better neural communication between the cortex and cerebellum, leading to better language function. Supplementary analyses revealed that MCP FA was significantly correlated with FA of multiple infratentorial tracts, including the inferior and SCPs, ML and CPs. These inter-tract associations reinforce MCP's role as a major cerebellar relay hub, integrating sensorimotor and cognitive information from widespread brain regions. Given its connectivity, disruptions in cerebro-cerebellar pathways may contribute to language deficits, further underscoring MCP's importance in PSA. This aligns with our findings on naming and word-finding performance, where greater MCP integrity may facilitate more efficient cerebellar contributions to semantic retrieval, consistent with the cerebellum's established role in non-motor language functions discussed earlier.^9,10,39^

The ICP interfaces the spinal cord and medulla oblongata with the cerebellum, with afferent fibres passing lateral to the spinal tract of the trigeminal nerve to enter the cerebellum.^40^ It receives proprioceptive information from the spinal cord and inputs from nuclei in the medulla, participating in the cerebellum's role of balance and fine-tuning movements.^41^ As the left side of the cerebellum seems to be associated with timing and sequencing of precise motor planning, the L-ICP, which primarily conveys ipsilateral proprioceptive information from the spinal cord, likely plays a crucial role in supporting the temporal coordination of motor tasks such as speech production.^9-11,40,41^ This functional alignment may explain why the L-ICP demonstrates a stronger association with language function than the right ICP. We speculate that overall, higher structural white matter integrity in the L-ICP may correspond with enhanced connectivity between the aforementioned regions, leading to improved integration of proprioceptive information and movement, contributing to better language capabilities via smoothing of speech production.^40,41^

In our study, the FA of the region of the L-CST below the tentorium was found to be correlated with SS and AQ. The CST, part of the pyramidal tract, is one of the major neuronal pathways connecting the spinal cord and motor regions of the cortex.^42^ It plays a major role in cortical control of spinal cord activity and thus is the principal motor pathway for voluntary movements.^39,42^ Since motor nuclei needed for phonation and articulation are located from the pons down to the spinal cord's lumbar portion, integrity of the CST is heavily associated with speech production.^35,42^ The left hemisphere houses critical language areas such as Broca's area, which formulates articulatory information to be executed predominantly by the left motor cortex.^22^ The L-CST originates from the left motor cortex, serving as a critical pathway for transmitting motor commands from these language regions to the spinal cord. Before crossing at the medulla, the L-CST remains ipsilateral to the left motor cortex, showing a direct connection for executing motor commands essential to speech production.^42^ This anatomical basis, coupled with the fact that our participants mainly had cortical lesions in the left perisylvian area, likely explains its stronger association with speech-related functions compared to the right CST. As higher FA indicates less damage to the structure of the white matter tracts of the infratentorial L-CST, we postulate a more robust pathway between cortical brain regions and the motor nuclei recruited in voluntary generation of speech.^39,42^

When considering the statistical significance of the left arcuate lesion volume's association with WAB-R scores in our sample, it appears that the structural integrities of the MCP, L-ICP and L-CST also show notable correlation with aphasia severity following stroke. Despite the arcuate fasciculus' well-documented role in language processing and aphasia, drawing inferences from our data, FA of the infratentorial white matter tracts hold similar predictive potential.^23,43-45^ This likely stems from the distinct nature of these measures (arcuate lesion volume and FA). Lesion overlap provides a binary measure of structural damage but does not account for surviving fibre integrity, secondary degeneration or compensatory plasticity. In contrast, FA offers a more continuous and functionally relevant assessment of white matter microstructure, making it more sensitive to connectivity efficiency.^19,20,23^ This may explain why MCP FA exhibited stronger and more widespread correlations with language function, whereas AF lesion overlap was more limited. While FA of the AF could offer a more direct assessment of its integrity, supratentorial lesions often complicate registration and segmentation. In contrast, cerebellar white matter, including the MCP, is typically more preserved, allowing for more reliable FA measurements in this cohort. However, this does not necessarily mean MCP FA is inherently a stronger predictor than AF FA, but rather that its greater measurement consistency may have contributed to its stronger observed associations. This highlights the importance of considering both methodological and anatomical factors when interpreting white matter-language relationships. The statistical significance of univariate linear regression models using the MCP, L-ICP and L-CST compared to that of the left arcuate lesion volume again suggests that these infratentorial brain regions can provide a substantial and consistent basis for examining WAB-R scores after a stroke.

An important observation is the change in significance after controlling for arcuate fasciculus damage. While MCP remains significant, L-CST and L-ICP become non-significant, suggesting their associations with language function reflect cerebellar contributions to cerebral damage rather than independent roles. However, this remains clinically relevant, as L-CST and L-ICP integrity may serve as indirect markers of cerebral damage, offering an alternative means to assess stroke-related structural deficits when supratentorial measures are less reliable. Notably, our supplementary analyses found that L-CST FA exhibited a significant negative correlation with arcuate fasciculus lesion volume, further supporting the idea that infratentorial white matter integrity may be indirectly affected by supratentorial stroke damage.

These findings not only underscore the complexity of brain networks involved in language function, but highlight a critical insight: the neuroanatomical substrates of aphasia extend beyond conventional language circuits, possibly incorporating a wider range of mechanisms that include cerebellar and corticospinal involvement.^46-48^ The predictive power and correlational strength of infratentorial regions also provide a possible solution to the methodological challenges posed by extensive cortical damage in PSA patients. This focus on such regions could lead to better feasibility and ease of automation of evaluating PSA, especially when compared to the investigation of supratentorial areas. Future research should further explore these infratentorial white matter regions to develop more robust and clinically applicable biomarkers for PSA, potentially offering new avenues for diagnosis and treatment planning in cases where traditional supratentorial analyses are limited.

## Limitations

This study is subject to certain limitations. Even though the participants primarily experienced damage to the left superior longitudinal fasciculus, the differences in individual stroke lesions warrant a careful, holistic interpretation of results. This heterogeneity of stroke lesions among participants suggests the need for a larger sample size to better account for this variability. A larger cohort would allow for a more nuanced understanding of how different lesion characteristics, such as size and location, influence the relationship between white matter integrity and language outcomes.

However, given the significance of the correlations between white matter integrity and aphasia severity in infratentorial white matter regions, including both cerebellar pathways and the CST, we believe our findings provide valuable insights into the broader neural network involved in PSA. These results highlight the importance of considering both cerebellar and corticospinal pathways in understanding language deficits following stroke.

Another limitation of this study is the confined scope of our analyses to infratentorial white matter integrity (i.e. FA), excluding supratentorial white matter integrity from our investigation. This approach was necessitated by the prevalence of lesions in supratentorial areas, which often impede accurate delineation and quantification of white matter integrity in traditional language-associated regions. The relative preservation of infratentorial structures in PSA patients provided a unique opportunity to investigate potential biomarkers and neural correlates that are less confounded by stroke-induced structural changes, offering methodological advantages in terms of feasibility and reliability. However, this focus potentially overlooks the complex interplay between supratentorial and infratentorial networks in language processing. Even though our investigation of the relationship between the left arcuate fasciculus lesion volume and language function allows an exploration of the supratentorial region's influence, without a direct analysis between the arcuate fasciculus and infratentorial regions, we cannot completely mitigate this concern.

While our preprocessing approach follows widely used methods, including the JHU white matter atlas and FSL DTI preprocessing pipeline, it does not incorporate cerebellum-specific templates and registration methods. Although our approach can help ensure compatibility with common research and clinical workflows, templates such as SUIT or CERES may offer greater anatomical precision. Future studies could explore specialized cerebellar preprocessing to improve segmentation accuracy and optimize infratentorial analyses.

Additionally, the exclusion of diffusion imaging metrics such as AD and RD limits our ability to understand microstructural mechanisms of cerebellar contributions to language. Future studies should include these in their analyses in order to gain insight into axonal and myelin damage, which could further elucidate the role of cerebellar white matter tracts in PSA.

## Conclusion

Our study provides novel insights into the role of infratentorial white matter integrity, particularly in the MCP, L-ICP and L-CST, in PSA severity. These regions demonstrated significant correlations with language performance and emerged as reliable predictors of aphasia severity, underscoring their potential as biomarkers. Importantly, by shifting focus to relatively preserved infratentorial structures, this approach circumvents challenges associated with cortical lesions, offering a more consistent framework for evaluating aphasia in patients with varying lesion profiles. The findings emphasize the involvement of cerebellar and corticospinal pathways in language processing, expanding traditional models that have predominantly focused on supratentorial regions. This expanded understanding may inform future clinical assessments and therapeutic interventions targeting not only cortical but also cerebellar and corticospinal pathways. Future research should explore the combined contributions of both infratentorial and supratentorial regions to offer a more comprehensive understanding of PSA mechanisms.

## Supplementary Material

fcaf174_Supplementary_Data

## Data Availability

The data that support the findings of this study are available from the corresponding author, upon reasonable request. The codes used for DTI analysis in this study have been included in the Supplementary material.

## References

[1] Franz SI . Review of Aphasia and Kindred Disorders of Speech, by H. Head. Am J Psychol. 1928;40:131–135.

[2] Skipper-Kallal LM, Lacey EH, Xing S, Turkeltaub PE. Right hemisphere remapping of naming functions depends on lesion size and location in poststroke aphasia. Neural Plast. 2017;2017:8740353. 28168061 10.1155/2017/8740353 PMC5266856

[3] Thye M, Mirman D. Relative contributions of lesion location and lesion size to predictions of varied language deficits in post-stroke aphasia. Neuroimage Clin. 2018;20:1129–1138. 30380520 10.1016/j.nicl.2018.10.017 PMC6205357

[4] Sul B, Lee KB, Hong BY, et al Association of lesion location with long-term recovery in post-stroke aphasia and language deficits. Front Neurol. 2019;10:776. 31396146 10.3389/fneur.2019.00776 PMC6668327

[5] Daria F, Elena P, Galina P, Olga M, Alina T, Vladislav B. The influence of lesion volume, cortex thickness, and lesion localization on chronic post-stroke aphasia severity. In: IEEE Symposium series on computational intelligence (SSCI). IEEE; 2019:541–549.

[6] Hickok G, Poeppel D. The cortical organization of speech processing. Nat Rev Neurosci. 2007;8(5):393–402. 17431404 10.1038/nrn2113

[7] Tourville JA, Guenther FH. The DIVA model: A neural theory of speech acquisition and production. Lang Cogn Process. 2011;26(7):952–981. 23667281 10.1080/01690960903498424 PMC3650855

[8] Fridriksson J, den Ouden DB, Hillis AE, et al Anatomy of aphasia revisited. Brain. 2018;141(3):848–862. 29360947 10.1093/brain/awx363 PMC5837461

[9] De Smet HJ, Paquier P, Verhoeven J, Mariën P. The cerebellum: Its role in language and related cognitive and affective functions. Brain Lang. 2013;127(3):334–342. 23333152 10.1016/j.bandl.2012.11.001

[10] Mariën P, Borgatti R. Language and the cerebellum. Handb Clin Neurol. 2018;154:181–202. 29903439 10.1016/B978-0-444-63956-1.00011-4

[11] Starowicz-Filip A, Chrobak AA, Moskała M, et al The role of the cerebellum in the regulation of language functions. Psychiatr Pol. 2017;51(4):661–671. 28987056 10.12740/PP/68547

[12] LeBel A., D’Mello AM. A seat at the (language) table: Incorporating the cerebellum into frameworks for language processing. Curr Opin Behav Sci. 2023;53:101310.

[13] Ackermann H . The contribution of the cerebellum to speech production and speech perception: Clinical and functional imaging data. Cerebellum. 2007;6:202–213. 17786816 10.1080/14734220701266742

[14] Heller SL, Heier LA, Watts R, et al Evidence of cerebral reorganization following perinatal stroke demonstrated with fMRI and DTI tractography. Clin Imaging. 2005;29(4):283–287. 15967322 10.1016/j.clinimag.2004.09.003

[15] Meinzer M, Mohammadi S, Kugel H, et al Integrity of the hippocampus and surrounding white matter is correlated with language training success in aphasia. Neuroimage. 2010;53(1):283–290. 20541018 10.1016/j.neuroimage.2010.06.004

[16] Friederici AD . White-matter pathways for speech and language processing. Handb Clin Neurol. 2015;129:177–186. 25726269 10.1016/B978-0-444-62630-1.00010-X

[17] Ivanova MV, Isaev DY, Dragoy OV, et al Diffusion-tensor imaging of major white matter tracts and their role in language processing in aphasia. Cortex. 2016;85:165–181. 27289586 10.1016/j.cortex.2016.04.019

[18] Xing S, Lacey EH, Skipper-Kallal LM, Zeng J, Turkeltaub PE. White matter correlates of auditory comprehension outcomes in chronic post-stroke aphasia. Front Neurol. 2017;8:54. 28275366 10.3389/fneur.2017.00054 PMC5319956

[19] Muir KW, Buchan A, von Kummer R, Rother J, Baron JC. Imaging of acute stroke. Lancet Neurol. 2006;5(9):755–768. 16914404 10.1016/S1474-4422(06)70545-2

[20] Sagi Y, Tavor I, Hofstetter S, Tzur-Moryosef S, Blumenfeld-Katzir T, Assaf Y. Learning in the fast lane: New insights into neuroplasticity. Neuron. 2012;73(6):1195–1203. 22445346 10.1016/j.neuron.2012.01.025

[21] Hosomi A, Nagakane Y, Yamada K, et al Assessment of arcuate fasciculus with diffusion-tensor tractography may predict the prognosis of aphasia in patients with left middle cerebral artery infarcts. Neuroradiology. 2009;51(9):549–555. 19434402 10.1007/s00234-009-0534-7

[22] Geva S, Correia MM, Warburton EA. Contributions of bilateral white matter to chronic aphasia symptoms as assessed by diffusion tensor MRI. Brain Lang. 2015;150:117–128. 26401977 10.1016/j.bandl.2015.09.001 PMC4669306

[23] Jang SH . Diffusion tensor imaging studies on arcuate fasciculus in stroke patients: A review. Front Hum Neurosci. 2013;7:749. 24198780 10.3389/fnhum.2013.00749 PMC3814569

[24] Lutkenhoff ES, Rosenberg M, Chiang J, et al Optimized brain extraction for pathological brains (optiBET). PLos One. 2014; 9(12):e115551. 25514672 10.1371/journal.pone.0115551 PMC4267825

[25] Pustina D, Coslett HB, Turkeltaub PE, Tustison N, Schwartz MF, Avants B. Automated segmentation of chronic stroke lesions using LINDA: Lesion identification with neighborhood data analysis. Hum Brain Mapp. 2016;37(4):1405–1421. 26756101 10.1002/hbm.23110 PMC4783237

[26] Avants BB, Tustison NJ, Song G, Cook PA, Klein A, Gee JC. A reproducible evaluation of ANTs similarity metric performance in brain image registration. Neuroimage. 2011;54(3):2033–2044. 20851191 10.1016/j.neuroimage.2010.09.025 PMC3065962

[27] Oguz I, Farzinfar M, Matsui J, et al DTIPrep: Quality control of diffusion-weighted images. Front Neuroinform. 2014;8:4. 24523693 10.3389/fninf.2014.00004 PMC3906573

[28] Smith SM . Fast robust automated brain extraction. Hum Brain Mapp. 2002;17(3):143–155. 12391568 10.1002/hbm.10062 PMC6871816

[29] Assaf Y, Pasternak O. Diffusion tensor imaging (DTI)-based white matter mapping in brain research: A review. J Mol Neurosci. 2008;34(1):51–61. 18157658 10.1007/s12031-007-0029-0

[30] Doughty C, Wang J, Feng W, Hackney D, Pani E, Schlaug G. Detection and predictive value of fractional anisotropy changes of the corticospinal tract in the acute phase of a stroke. Stroke. 2016;47(6):1520–1526. 27217504 10.1161/STROKEAHA.115.012088 PMC4959886

[31] Wan CY, Zheng X, Marchina S, Norton A, Schlaug G. Intensive therapy induces contralateral white matter changes in chronic stroke patients with Broca’s aphasia. Brain Lang. 2014;136:1–7. 25041868 10.1016/j.bandl.2014.03.011 PMC4425280

[32] Williamson J, Nyenhuis D, Stebbins GT, et al Regional differences in relationships between apparent white matter integrity, cognition, and mood in patients with ischemic stroke. J Clin Exp Neuropsychol. 2010;32(7):673–681. 20155558 10.1080/13803390903427406

[33] Magnin E, Cattin F, Vandel P, Galmiche J, Moulin T, Rumbach L. Fractional anisotropy in three variants of primary progressive aphasia. Eur Neurol. 2012;68(4):229–233. 22964895 10.1159/000339947

[34] Morales H, Tomsick T. Middle cerebellar peduncles: Magnetic resonance imaging and pathophysiologic correlate. World J Radiol. 2015;7(12):438–447. 26751508 10.4329/wjr.v7.i12.438 PMC4697118

[35] Tremblay P, Deschamps I, Gracco VL. Neurobiology of speech production: A motor control perspective. In: Hickok G, Small SL, eds. Neurobiology of language. eds. Academic Press; 2016:741–750.

[36] Skipper JI, Lametti DR. Speech perception under the tent: A domain-general predictive role for the cerebellum. J Cogn Neurosci. 2021;33(8):1517–1534. 34496370 10.1162/jocn_a_01729

[37] Hertrich I, Mathiak K, Ackermann H. The role of the cerebellum in speech perception and language comprehension. In: Mariën P, Manto M, eds. The linguistic cerebellum: Academic Press; 2016:33–50.

[38] Vias C, Dick AS. Cerebellar contributions to language in typical and atypical development: A review. Dev Neuropsychol. 2017;42(6):404–421. 28885046 10.1080/87565641.2017.1334783 PMC6232854

[39] Armstrong RA . The structure of the human brain as revealed in six histological sections. In: Martin CR, Preedy VR, Rajendram R, eds. Diagnosis, management and modeling of neurodevelopmental disorders. Academic Press; 2021:3–11.

[40] Naidich TP, Stein EG, Lento PA, Kleinman GM, Elizabeth Fowkes M, Carpenter DM. Chapter 16 - Cerebellum. In: Naidich TP, Castillo M, Cha S, Smirniotopoulos JG, eds. Imaging of the Brain. W.B. Saunders; 2013:328–347.

[41] Jang S, Kwon H. Connectivity of Inferior cerebellar peduncle in the human brain: A diffusion tensor imaging study. Neural Network World. 2016;26:439–447.

[42] Welniarz Q, Dusart I, Roze E. The corticospinal tract: Evolution, development, and human disorders. Dev Neurobiol. 2017;77(7):810–829. 27706924 10.1002/dneu.22455

[43] Kümmerer D, Hartwigsen G, Kellmeyer P, et al Damage to ventral and dorsal language pathways in acute aphasia. Brain. 2013;136(2):619–629. 23378217 10.1093/brain/aws354 PMC3572927

[44] Marchina S, Zhu LL, Norton A, Zipse L, Wan CY, Schlaug G. Impairment of speech production predicted by lesion load of the left arcuate fasciculus. Stroke. 2011;42(8):2251–2256. 21719773 10.1161/STROKEAHA.110.606103 PMC3167233

[45] Fridriksson J, Guo D, Fillmore P, Holland A, Rorden C. Damage to the anterior arcuate fasciculus predicts non-fluent speech production in aphasia. Brain. 2013;136(11):3451–3460. 24131592 10.1093/brain/awt267 PMC3808690

[46] Dronkers NF, Redfern BB, Knight RT. The neural architecture of language disorders. In: Gazzaniga MS, ed. The new cognitive neurosciences, 2nd ed. MIT Press; 2000:949–960.

[47] Dronkers NF, Plaisant O, Iba-Zizen MT, Cabanis EA. Paul Broca’s historic cases: High-resolution MR imaging of the brains of Leborgne and Lelong. Brain. 2007;130(5):1432–1441. 17405763 10.1093/brain/awm042

[48] Zheng ZS, Wang KX, Millan H, et al Impact of neuromodulation on post-stroke aphasia: a multimodal randomized controlled study. medRxiv. [Preprint]

