# Supplementary material for: Infratentorial white matter integrity as a potential biomarker for post-stroke aphasia
Source: Brain Commun. 2025 May 6;7(3):fcaf174. doi: 10.1093/braincomms/fcaf174 (PMC12079383; doi:10.1093/braincomms/fcaf174)
Supplement: fcaf174_Supplementary_Data [file fcaf174_supplementary_data.docx]

**Supplementary Materials**

**Supplemental Methods**

***Arcuate Lesion Calculation***

Given the critical role of the arcuate fasciculus in language processing, the extent of damage to this tract likely has a significant impact on the severity of PSA.^1-4^ Therefore, we opted to calculate the overlap between each patient’s stroke lesion and their arcuate fasciculus to better understand its impact on their condition, as well as compare its effect on PSA against that of infratentorial white matter tracts.

A tractography-based atlas of the arcuate fasciculus, in standard MNI space, was originally obtained from NatBrainLab (formerly available at [https://www.natbrainlab.co.uk/atlas-maps], though the website is no longer accessible). To ensure reproducibility, we have provided a copy of the atlas at [https://neurovault.org/collections/19848/], while crediting NatBrainLab as the original provider. The atlas was then binarized and separated into left and right segments to facilitate the subsequent overlap analysis. Each participant’s T1-weighted MRI scan was standardized to the MNI space and binarized as well. Lesion masks were normalized to standard space using transformation matrices derived from high-resolution T1-weighted images, ensuring alignment while preserving the spatial integrity of lesion boundaries.^5, 6^ Since lesion masks are binary representations of stroke lesions, maintaining their precise shape is essential for accurate analysis. Stroke-related structural displacements can affect registration, and we found that nonlinear transformations introduced significant distortions, altering lesion size and location. By using linear registration, we minimized these distortions and ensured that lesion masks remained spatially consistent for group-level analyses, allowing for reliable intersection with atlas-defined ROIs, such as the arcuate fasciculus.

Binarized lesion mask were then intersected with the binarized arcuate fasciculus ROI using the fslmaths command. This procedure generated new images for each subject, representing the overlap between the lesion and the left and right arcuate segments, thereby isolating the affected portions of the tract. The volume of overlap (arcuate lesion volume), indicative of the extent to which the arcuate fasciculus was compromised by the lesion, was quantified using the fslstats command.

***Statistical Analysis***

We analyzed Spearman’s correlations between left arcuate lesion volume and WAB-R scores, as most participants had lesions affecting this canonical language structure. While white matter integrity measures like FA could be informative, especially in infratentorial regions (relatively spared in PSA), the extensive lesions in our cohort precluded reliable brain registration in regions above the tentorium. This limitation necessitated that our supratentorial focus be on lesion volume. The Benjamini-Hochberg method (FDR = 0.05) was utilized for multiple comparisons correction. Based on these findings, univariate linear regression analyses were conducted to pinpoint which of the WAB-R scores were reliably predicted by arcuate lesion volume.

**Supplemental Results**

We found that after multiple comparisons correction, the left arcuate lesion volume correlated with AQ (r_s_ = -0.352, p = 0.014) and REP (r_s_ = -0.433, p = 0.002). Scatterplots of the results are shown in Supplementary Figure 1. Additionally via linear regression (Supplementary Table 1), the left arcuate lesion volume showed statistical significance as a predictor of AQ (t_53_ = -2.599, p = 0.013), SS (t_53_ = -2.198, p = 0.033), NW (t_53_ = -2.171, p = 0.035), and REP (t_53_ = -3.366, p = 0.002). When analyzing correlations between average FA of the twelve infratentorial white matter tracts and the left arcuate lesion volume, we found that MCP was significantly correlated with the following: PCT (p < 0.001), R-CST (p = 0.044), L-CST (p < 0.001), R-ML (p < 0.001), L-ML (p < 0.001), R-ICP (p < 0.001), L-ICP (p < 0.001), R-SCP (p < 0.001), L-SCP (p < 0.001), R-CP (p < 0.001), L-CP (p < 0.001). L-CST was found to be correlated with Left Arcuate Lesion Overlap (P < 0.001).

**Supplementary Table 1. Left Arcuate Lesion Linear Regression Results**

| Dependent Variable |  | Independent Variable | | | t | p-value |  |
| --- | --- | --- | --- | --- | --- | --- | --- |
| AQ |  | Left Arcuate Lesion Volume | | | -2.599 | 0.013 | * |
|  |  |  | | |  |  |  |
| SS |  | Left Arcuate Lesion Volume | | | -2.198 | 0.033 | * |
|  | | | | | | | |
| AVC |  | Left Arcuate Lesion Volume | | | -1.938 | 0.059 |  |
|  |  |  |  |  |  |  |  |
| NW |  | Left Arcuate Lesion Volume | | | -2.171 | 0.035 | * |
|  | | | | | | | |
| REP |  | Left Arcuate Lesion Volume | | | -3.366 | 0.002 | * |

Supplementary Table 1 shows the results of univariate linear regression models (N=55) using Left Arcuate Lesion Volume as the independent variable, and WAB-R subscores as the dependent variables.

**Supplementary Figure 1. Left Arcuate Lesion Volume Scatterplots**


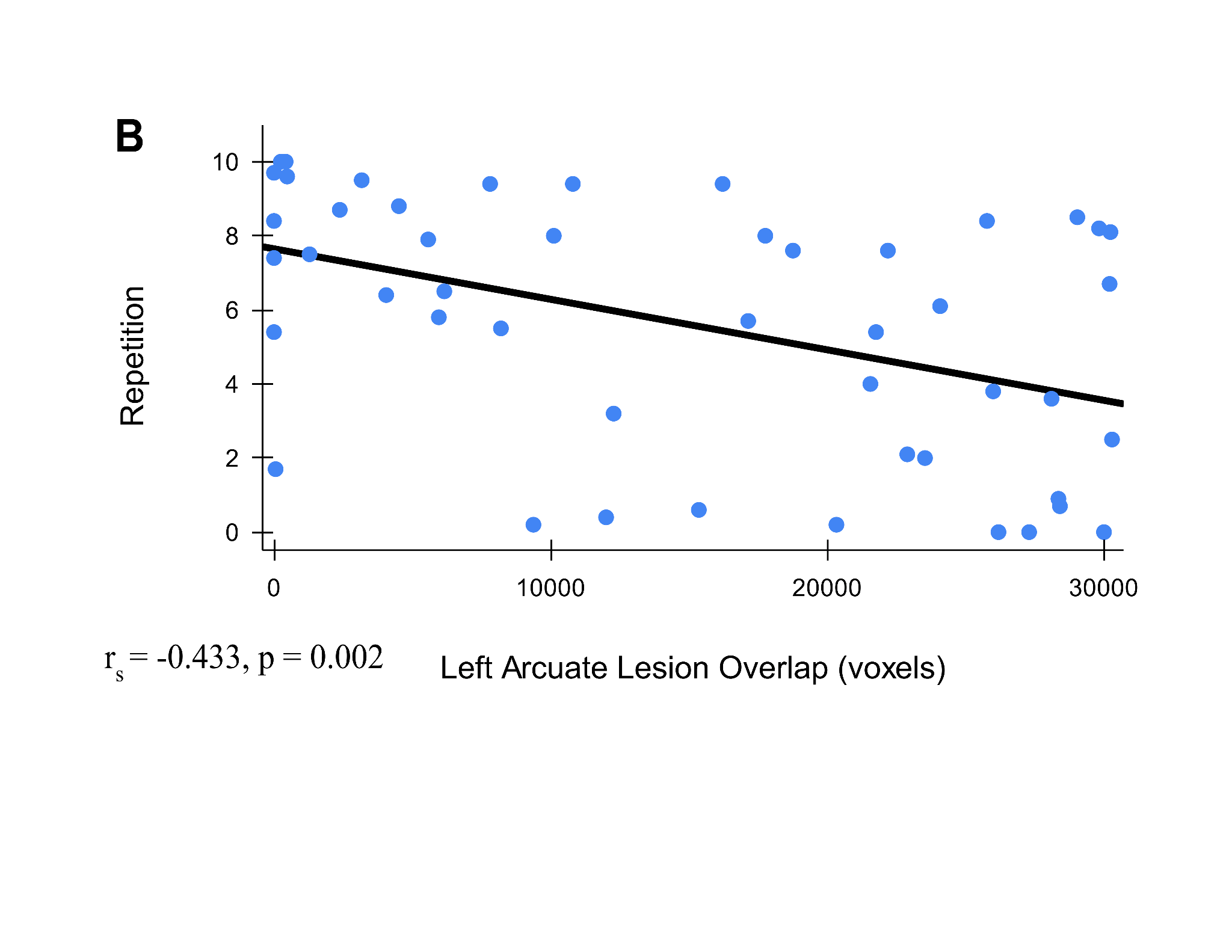

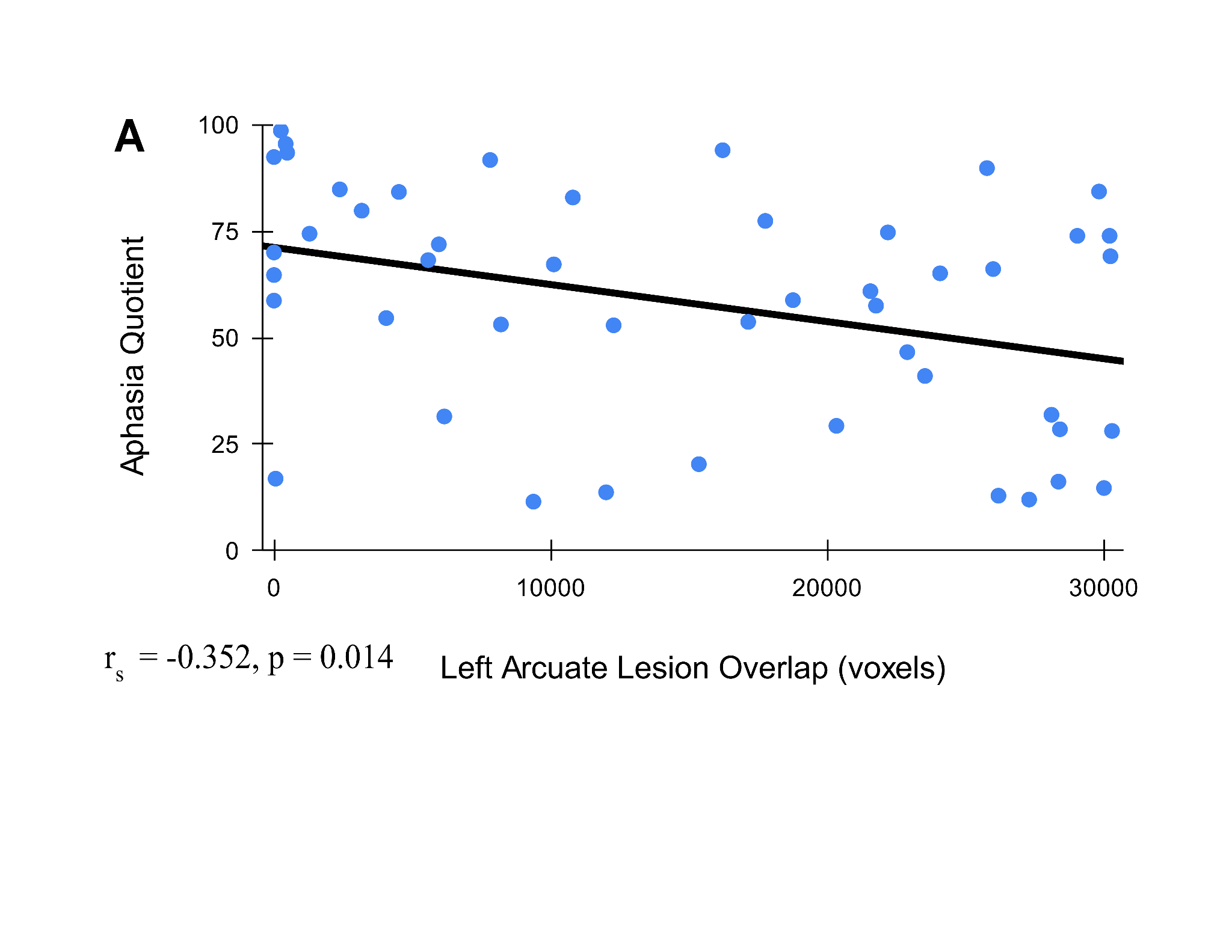


**(A-B)** Scatterplots indicating the Spearman’s correlation between Left Arcuate Lesion Volume (voxels) and the following Western Aphasia Battery-Revised scores : Aphasia Quotient, Repetition (N=55). Plots are shown for **(A)** aphasia quotient and **(B)** repetition. Each data point represents an individual participant.

Supplementary Table 2. White Matter Tract & Left Arcuate Lesion Correlations

| **Variable** |  | | Middle Cerebellar Peduncle | | Pontine Crossing Tract | | Right Corticospinal Tract | | Left Corticospinal Tract | | Right Medial Lemniscus | | Left Medial Lemniscus | | Right Inferior Cerebellar Peduncle | | Left Inferior Cerebellar Peduncle | | Right Superior Cerebellar Peduncle | | Left Superior Cerebellar Peduncle | | Right Cerebral Peduncle | | Left Cerebral Peduncle | |
| --- | --- | --- | --- | --- | --- | --- | --- | --- | --- | --- | --- | --- | --- | --- | --- | --- | --- | --- | --- | --- | --- | --- | --- | --- | --- | --- |
| Middle Cerebellar Peduncle | Spearman's rho |  | — |  |  |  |  |  |  |  |  |  |  |  |  |  |  |  |  |  |  |  |  |  |  |  |
|  | p-value |  | — |  |  |  |  |  |  |  |  |  |  |  |  |  |  |  |  |  |  |  |  |  |  |  |
| Pontine Crossing Tract | Spearman's rho |  | 0.498 | *** | — |  |  |  |  |  |  |  |  |  |  |  |  |  |  |  |  |  |  |  |  |  |
|  | p-value |  | < .001 |  | — |  |  |  |  |  |  |  |  |  |  |  |  |  |  |  |  |  |  |  |  |  |
| Right Corticospinal Tract | Spearman's rho |  | 0.279 | * | 0.326 | * | — |  |  |  |  |  |  |  |  |  |  |  |  |  |  |  |  |  |  |  |
|  | p-value |  | 0.044 |  | 0.017 |  | — |  |  |  |  |  |  |  |  |  |  |  |  |  |  |  |  |  |  |  |
| Left Corticospinal Tract | Spearman's rho |  | 0.492 | *** | 0.219 |  | 0.207 |  | — |  |  |  |  |  |  |  |  |  |  |  |  |  |  |  |  |  |
|  | p-value |  | < .001 |  | 0.115 |  | 0.137 |  | — |  |  |  |  |  |  |  |  |  |  |  |  |  |  |  |  |  |
| Right Medial Lemniscus | Spearman's rho |  | 0.699 | *** | 0.413 | ** | 0.372 | ** | 0.385 | ** | — |  |  |  |  |  |  |  |  |  |  |  |  |  |  |  |
|  | p-value |  | < .001 |  | 0.002 |  | 0.006 |  | 0.005 |  | — |  |  |  |  |  |  |  |  |  |  |  |  |  |  |  |
| Left Medial Lemniscus | Spearman's rho |  | 0.647 | *** | 0.493 | *** | 0.493 | *** | 0.406 | ** | 0.831 | *** | — |  |  |  |  |  |  |  |  |  |  |  |  |  |
|  | p-value |  | < .001 |  | < .001 |  | < .001 |  | 0.003 |  | < .001 |  | — |  |  |  |  |  |  |  |  |  |  |  |  |  |
| Right Inferior Cerebellar Peduncle | Spearman's rho |  | 0.676 | *** | 0.36 | ** | 0.49 | *** | 0.332 | * | 0.677 | *** | 0.69 | *** | — |  |  |  |  |  |  |  |  |  |  |  |
|  | p-value |  | < .001 |  | 0.008 |  | < .001 |  | 0.015 |  | < .001 |  | < .001 |  | — |  |  |  |  |  |  |  |  |  |  |  |
| Left Inferior Cerebellar Peduncle | Spearman's rho |  | 0.7 | *** | 0.383 | ** | 0.396 | ** | 0.471 | *** | 0.751 | *** | 0.815 | *** | 0.709 | *** | — |  |  |  |  |  |  |  |  |  |
|  | p-value |  | < .001 |  | 0.005 |  | 0.004 |  | < .001 |  | < .001 |  | < .001 |  | < .001 |  | — |  |  |  |  |  |  |  |  |  |
| Right Superior Cerebellar Peduncle | Spearman's rho |  | 0.749 | *** | 0.39 | ** | 0.196 |  | 0.478 | *** | 0.592 | *** | 0.59 | *** | 0.561 | *** | 0.653 | *** | — |  |  |  |  |  |  |  |
|  | p-value |  | < .001 |  | 0.004 |  | 0.16 |  | < .001 |  | < .001 |  | < .001 |  | < .001 |  | < .001 |  | — |  |  |  |  |  |  |  |
| Left Superior Cerebellar Peduncle | Spearman's rho |  | 0.814 | *** | 0.363 | ** | 0.184 |  | 0.565 | *** | 0.623 | *** | 0.553 | *** | 0.568 | *** | 0.597 | *** | 0.815 | *** | — |  |  |  |  |  |
|  | p-value |  | < .001 |  | 0.008 |  | 0.187 |  | < .001 |  | < .001 |  | < .001 |  | < .001 |  | < .001 |  | < .001 |  | — |  |  |  |  |  |
| Right Cerebral Peduncle | Spearman's rho |  | 0.717 | *** | 0.285 | * | 0.373 | ** | 0.416 | ** | 0.646 | *** | 0.655 | *** | 0.558 | *** | 0.712 | *** | 0.646 | *** | 0.698 | *** | — |  |  |  |
|  | p-value |  | < .001 |  | 0.039 |  | 0.006 |  | 0.002 |  | < .001 |  | < .001 |  | < .001 |  | < .001 |  | < .001 |  | < .001 |  | — |  |  |  |
| Left Cerebral Peduncle | Spearman's rho |  | 0.588 | *** | 0.191 |  | 0.202 |  | 0.74 | *** | 0.471 | *** | 0.541 | *** | 0.395 | ** | 0.604 | *** | 0.645 | *** | 0.616 | *** | 0.582 | *** | — |  |
|  | p-value |  | < .001 |  | 0.169 |  | 0.146 |  | < .001 |  | < .001 |  | < .001 |  | 0.004 |  | < .001 |  | < .001 |  | < .001 |  | < .001 |  | — |  |
| Left Arcuate Overlap Volume | Spearman's rho |  | -0.338 | * | -0.127 |  | -0.166 |  | -0.469 | *** | -0.227 |  | -0.215 |  | -0.209 |  | -0.102 |  | -0.218 |  | -0.416 | ** | -0.17 |  | -0.284 |  |
|  | p-value |  | 0.019 |  | 0.39 |  | 0.259 |  | < .001 |  | 0.121 |  | 0.142 |  | 0.154 |  | 0.491 |  | 0.136 |  | 0.003 |  | 0.249 |  | 0.051 |  |
| * Statistically Significant |  |  |  |  |  |  |  |  |  |  |  |  |  |  |  |  |  |  |  |  |  |  |  |  |  |  |

Supplementary Table 2 shows correlations between the average FA’s of all twelve infratentorial white matter tracts as well as the Left Arcuate Lesion Volume.

**Supplementary References**

1. Skipper-Kallal LM, Lacey EH, Xing S, Turkeltaub PE. Right hemisphere remapping of

naming functions depends on lesion size and location in poststroke aphasia. *Neural Plast.*

2017;2017:8740353. doi:10.1155/2017/8740353

2. Thye M, Mirman D. Relative contributions of lesion location and lesion size to predictions

of varied language deficits in post-stroke aphasia. *Neuroimage Clin*. 2018;20:1129-1138.

doi:10.1016/j.nicl.2018.10.017

3. Sul B, Lee KB, Hong BY, et al. Association of lesion location with long-term recovery in

post-stroke aphasia and language deficits. *Front Neurol.* 2019;10:77.

doi:10.3389/fneur.2019.00776

4. Daria F, Elena P, Galina P, Olga M, Alina T, Vladislav B. The influence of lesion volume,

cortex thickness, and lesion localization on chronic post-stroke aphasia severity. *In:*

*IEEE Symposium Series on Computational Intelligence (SSCI).* Xiamen, China; 2019

2019:541-549. doi:10.1109/SSCI44817.2019.9002800

5. Lutkenhoff ES, Rosenberg M, Chiang J, et al. Optimized brain extraction for pathological

brains (optiBET). *PLoS One*. 2014;9(12). doi:10.1371/journal.pone.0115551

6. Smith SM. Fast robust automated brain extraction. *Hum Brain Mapp*. 2002;17(3):143-155.

doi:10.1002/hbm.10062

**DTI Analysis (Bash scripts)**

#!/bin/sh

########################################################################

fsl_anat provides a general pipeline for processing anatomical images ###

# For more information, see https://fsl.fmrib.ox.ac.uk/fsl/fslwiki/fsl_anat #

########################################################################

fsl_anat --noreg --nononlinreg --noseg --nosubcortseg -o T1_struct -i ./co20*mprage*.nii*

#Brain Extraction

sh ./optiBET.sh -i ${i}/T1_struct.anat/T1_biascorr.nii.gz

## Tissue Segmentation ##

fast ${i}/T1_struct.anat/T1_biascorr_optiBET_brain.nii.gz

#extract 1st volume (nodif)

fslroi ${i}/DTIPrep/dwi_QCed.nii.gz ${i}/nodif 0 1

#brain extract the B0 weighted image with no diffusion applied

bet ${i}/nodif ${i}/nodif_brain -f .25 -m

#Run dti_fit

dtifit -k ${i}/DTIPrep/dwi_QCed.nii.gz -m ${i}/nodif_brain_mask -r ${i}/DTIPrep/dwi_QCed.bvec -b ${i}/DTIPrep/dwi_QCed.bval -o ${i}/dti_stats/dti

#This creates a radial diffusivity image, another type of diffusion metric#

#fslmaths ${i}/dti_stats/dti_L2.nii.gz -add ${i}/dti_stats/dti_L3.nii.gz -div 2 ${i}/dti_stats/dti_RD

#Add thresholding to voxels

fslmaths ${i}/dti_stats/dti_FA.nii.gz -uthr 1 ${i}/dti_stats/dti_FA_uthr1

#####################################################################

MAKE SURE TO HAVE NODIF_BRAIN EXTRACTED ALREADY

#####################################################################

################################################# ####################

### xfm between nodif and struct (T1_optiBET)

################################################# ####################

flirt -in ${i}/nodif_brain.nii.gz -ref ${i}/T1_struct.anat/T1_biascorr_optiBET_brain.nii.gz -dof 6 -cost mutualinfo -omat ${i}/nodif2struct.mat

#Creates the inverse transformation matrix#

convert_xfm -omat ${i}/struct2nodif.mat -inverse ${i}/nodif2struct.mat

### Apply xfms to white matter mask (pve_2) ##

flirt -in ${i}/T1_struct.anat/T1_biascorr_optiBET_brain_pve_2.nii.gz -ref ${i}/nodif_brain.nii.gz -applyxfm -init ${i}/struct2nodif.mat -interp nearestneighbour -out ${i}/FAST/WM_seg_in_diff.nii.gz

### fslreorient2std ###

fslreorient2std ${i}/FAST/WM_seg_in_diff.nii.gz ${i}/FAST/WM_seg_in_diff_fsloriented.nii.gz

###########################################

####Creating linear and nonlinear xfms ####

###########################################

####Step 1: Linear xfm from dti_FA to FMRIB58_FA ####

flirt -ref ${FSLDIR}/data/standard/FMRIB58_FA_1mm -in ${i}/dti_stats/dti_FA_fslreoriented.nii.gz -omat ${i}/affine_xfm_fslreoriented.mat

#fnirt --in=${i}/dti_stats/dti_FA.nii.gz --aff=${i}/affine_xfm.mat --inmask=${i}/T1_lesion_mask_inverted_in_diff.nii.gz --cout=${i}/nonlinear_xfm_lesion_masked --config=FA_2_FMRIB58_1mm --iout=${i}/nonlinear_xfm_lesion_masked_brain

####Step 2: nonlinear xfm from dti_FA to FMRIB58_FA ####

fnirt --in=${i}/dti_stats/dti_FA_fslreoriented.nii.gz --aff=${i}/affine_xfm_fslreoriented.mat --cout=${i}/nonlinear_xfm_fslreoriented --config=FA_2_FMRIB58_1mm #--iout=${i}/nonlinear_xfm

####Step 3: inverse nonlinear xfm--stand2dti_warp ####

invwarp --ref=${i}/dti_stats/dti_FA_fslreoriented.nii.gz --warp=${i}/nonlinear_xfm_fslreoriented --out=${i}/stand2dti_warp_fslreoriented

#################################

####Incorporating Lesion Mask####

#################################

#Step 1: Invert the lesion mask and put it in diff space

#fslmaths ${i}/T1_struct.anat/T1_optiBET_brain_mask.nii.gz -sub ${i}/T1_struct.anat/linda/Prediction3_native.nii.gz -bin ${i}/T1_struct.anat/linda/T1_lesion_mask_inverted.nii.gz

#fslmaths ${i}/T1_struct.anat/T1_optiBET_brain_mask.nii.gz -sub ${i}/T1_struct.anat/T1_optiBET_brain_mask_kw.nii.gz -bin ${i}/T1_struct.anat/linda/T1_lesion_mask_inverted.nii.gz

#flirt -in ${i}/T1_struct.anat/linda/T1_lesion_mask_inverted.nii.gz -ref ${i}/nodif_brain.nii.gz -applyxfm -init ${i}/struct2nodif.mat -interp nearestneighbour -out ${i}/T1_struct.anat/linda/T1_lesion_mask_inverted_in_diff

#Step 2: Running flirt, registering subjects' FA to standard FA map (already run)

#flirt -ref ${FSLDIR}/data/standard/FMRIB58_FA_1mm -in ${i}/dti_stats/dti_FA.nii.gz -omat ${i}/affine_xfm.mat

#Step 3: Running fnirt, supplying an inverted lesion mask in diff space (0's in the lesion, 1's elsewhere)

#fnirt --in=${i}/dti_stats/dti_FA.nii.gz --aff=${i}/affine_xfm.mat --inmask=${i}/T1_struct.anat/linda/T1_lesion_mask_inverted_in_diff.nii.gz --cout=${i}/nonlinear_xfm_lesion_mask --config=FA_2_FMRIB58_1mm --iout=${i}/nonlinear_xfm_lesion_mask_brain

#Step 4: Inverse warp

#invwarp --ref=${i}/dti_stats/dti_FA.nii.gz --warp=${i}/nonlinear_xfm_lesion_mask --out=${i}/stand2dti_warp_lesion_mask

#!/bin/sh

#JHU 48 WM Labels#

for m in 01 02 03 04 05 06 07 08 09 10 11 12 13 14 15 16 17 18 19 20 21 22 23 24 25 26 27 28 29 30 31 32 33 34 35 36 37 38 39 40 41 42 43 44 45 46 47 48 JHU-ICBM-labels-1mm #JHU-ICBM-tracts-maxprob-thr25-1mm

do

### Apply transformation to 48 JHU Labels moving from standard to native FA space ###

#applywarp --ref=${i}/dti_stats/dti_FA.nii.gz --in=../Aphasia/f-tDCS/ROIs/JHU_labels/1mm/${m}.nii.gz --warp=${i}/stand2dti_warp --out=${i}/dti_stats/${m}_in_FA --interp=nn

#applywarp --ref=${i}/dti_stats/dti_FA.nii.gz --in=../Aphasia/f-tDCS/ROIs/JHU_labels/1mm/JHU-ICBM-labels-1mm.nii.gz --warp=${i}/stand2dti_warp --out=${i}/dti_stats/JHU-ICBM-labels-1mm_in_FA --interp=nn

applywarp --ref=${i}/dti_stats/dti_FA_fslreoriented.nii.gz --in=../Aphasia/f-tDCS/ROIs/JHU_labels/1mm/${m}.nii.gz --warp=${i}/stand2dti_warp_fslreoriented --out=${i}/dti_stats/${m}_in_FA_fslreoriented --interp=nn

### Apply JHU tracts ###

#applywarp --ref=${i}/dti_stats/dti_FA_fslreoriented.nii.gz --in=../Atlases/${m}.nii.gz --warp=${i}/stand2dti_warp_fslreoriented --out=${i}/dti_stats/${m}_in_FA_fslreoriented --interp=nn

### Apply split masks ###

#applywarp --ref=${i}/dti_stats/dti_FA_fslreoriented.nii.gz --in=Atlas/L_1_MCP_1mm.nii.gz --warp=${i}/stand2dti_warp_fslreoriented --out=${i}/dti_stats/L_1_MCP_in_FA_fslreoriented --interp=nn

#applywarp --ref=${i}/dti_stats/dti_FA_fslreoriented.nii.gz --in=Atlas/R_1_MCP_1mm.nii.gz --warp=${i}/stand2dti_warp_fslreoriented --out=${i}/dti_stats/R_1_MCP_in_FA_fslreoriented --interp=nn

#applywarp --ref=${i}/dti_stats/dti_FA_fslreoriented.nii.gz --in=Atlas/L_2_PCT_1mm.nii.gz --warp=${i}/stand2dti_warp_fslreoriented --out=${i}/dti_stats/L_2_PCT_in_FA_fslreoriented --interp=nn

#applywarp --ref=${i}/dti_stats/dti_FA_fslreoriented.nii.gz --in=Atlas/R_2_PCT_1mm.nii.gz --warp=${i}/stand2dti_warp_fslreoriented --out=${i}/dti_stats/R_2_PCT_in_FA_fslreoriented --interp=nn

###### Applywarp using lesion-masked warp ######

#applywarp --ref=${i}/dti_stats/dti_FA.nii.gz --in=ROIs/JHU_labels/1mm/${m}.nii.gz --warp=${i}/stand2dti_warp_lesion_mask --out=${i}/dti_stats/${m}_in_FA --interp=nn

#applywarp --ref=${i}/dti_stats/dti_FA.nii.gz --in=ROIs/JHU_labels/1mm/JHU-ICBM-labels-1mm.nii.gz --warp=${i}/stand2dti_warp_lesion_mask --out=${i}/dti_stats/JHU-ICBM-labels-1mm_in_FA_lesion_mask_warp --interp=nn

### OptiBet script available at https://montilab.psych.ucla.edu/fmri-wiki/optibet
